# Current status and job satisfaction of village doctors in western China

**DOI:** 10.1097/MD.0000000000016693

**Published:** 2019-08-09

**Authors:** Qi Zhang, Jiayan Chen, Min Yang, Jay Pan, Xiaoping Li, Lin Yue, Yuan Huang, Tao Mao, Cong Zhang, Xiao Ma

**Affiliations:** a West China School of Public Health, Sichuan University, Chengdu, Sichuan; b School of Public Health, Tongji Medical College, Huazhong University of Science and Technology, Wuhan, Hubei; c Business School, Sichuan University, Chengdu, Sichuan; d Department of Epidemiology and Biostatistics, Kunming Medical University, Kunming, Yunnan; e Jiangsu Provincial Center for Disease Control and Prevention, Nanjing, Jiangsu; f West China Second University Hospital, Sichuan University, Chengdu, Sichuan, China.

**Keywords:** current status, job satisfaction, village doctors, western China

## Abstract

To strengthen rural health services, the Chinese government has launched a series of policies to promote health workforce development. This study aims to understand the current status of village doctors and to explore the factors associated with village doctors’ job satisfaction in western China. It also attempts to provide references for further building capacities of village doctors and promoting the development of rural health service policy.

A multistage stratified sampling method was used to obtain data from a cross-sectional survey on village doctors across 2 provinces of western China during 2012 to 2013. Quantitative data were collected from village doctors face-to-face, through a self-administered questionnaire.

Among the 370 respondents, 225 (60.8%) aged 25 to 44 years, and 268 (72.4%) were covered by health insurance. Their income and working time calculated by workloads were higher than their self-report results. Being healthy, working fewer years, and having government funding and facilities were the positive factors toward their job satisfaction. Village doctors working with government-funded village clinics or facilities were more likely to feel satisfied.

Problems identified previously such as low income and lack of insurance, heavy workload and aging were not detected in our study. Instead, village doctors were better-paid and better-covered by social insurance than other local rural residents, with increased job satisfaction. Government policies should pay more attention to improving the quality of rural health services and the income and security system of village doctors, to maintain and increase their job satisfaction and work enthusiasm. Further experimental study could evaluate effects of government input to improve rural health human resources and system development.

## 1 Introduction

The Basic Medical Care System in rural China is a 3-tiered health service system including village clinics, township health centers, and county hospitals. Village doctors, as the primary health service providers in village clinics, play an important role in the rural healthcare system.^[1–4]^ In China, the history of village doctors could be dated back to “barefoot doctors” of the 1960s’, who received only short-time training and then provided primary medical and health services to villagers. Over decades, the team of village doctors has reached 0.901 million by 2017. They were working at 0.632 million village clinics in rural China,^[5]^ and their major duties included disease prevention, maternal and child healthcare, health promotion, and basic treatment of common and minor diseases.^[6–9]^

With the fast development of economy, China quickened its steps of urbanization, further shrinking the scale of rural areas.^[10]^ Such social development and changes have also impacted the 3-tiered health service system. For instance, the New Cooperative Medical Scheme (NCMS) initiated in 2003 has made substantial achievements in increasing the access to healthcare among rural residents.^[11]^ In addition, the Chinese central government launched the Health System Reform Plan in 2009 to establish and improve a basic public health service program that covered both urban and rural residents.^[12]^ These new reforms inevitably made changes to health services and created challenges for village doctors, which are particularly impeding in rural areas accommodating approximately 603.46 million people.^[13]^ Western China is characterized by inconvenient transportation, underdeveloped economy, dispersed population, and multiethnic society, and village doctors in the western regions might thus face more challenges than their eastern and central counterparts. Unfortunately, problems such as quality of health services and distribution of qualified health providers remain largely unresolved there.^[14]^

Early studies on barefoot doctors, which began from 1972, focused on their training and career development.^[15–19]^ Researchers shifted their attention later on to questions including the disparity of human resources, lack of education and training, relatively low pay, social security, and job satisfaction, as well as the low service capacity of village doctors.^[20,21]^ For example, village doctors were challenged with heavy workload, low income, and lack of social security in Guizhou,^[22]^ aging and lack of social protection in Hebei Province,^[23]^ insufficient number and low salaries and benefits in Changzhou.^[24]^ Based on these studies, main conclusions were drawn that village doctors in China were facing problems including aging, low income, heavy workload, and poor social security.

Despite these findings, however, some problems are marked. For instance, a number of the studies were conducted a long time ago, some even as early as the beginning of Health System Reform Plan in 2009. In addition, the conclusion of low income and heavy workload of village doctors had known reporting bias because they were mostly based on the income data and personal feeling toward working intensity self-reported by respondents. Measures such as strengthening primary health care facilities and raising qualification and income of village doctors are important in the health system reform of China.^[25]^ As a result, they may well reduce or partly solve problems such as low income and aging that were identified in previous studies. On the one hand, it is necessary to understand the development status of village doctors and assess possible effects of health system reform in recent years on workforce at the grass-root level in rural China. On the other hand, job satisfaction of primary health workers is actually affected by multifactors including personal and professional characteristics.^[26]^ Lower level job satisfaction will jeopardize work motivation, to say the least, and may even impact primary health work negatively as a whole.^[27]^ While studying the status quo, job satisfaction should be given attention to.

Western China covers 686 million km^2^ in area and occupies 72% of China's territory.^[28]^ Altogether 380 million people live in this region, taking up approximately 29% of the total population of China.^[29]^ This region is also home to 44 ethnic minority groups that represent over 70% of the 55 ethnic minority groups of the country.^[30]^ In terms of economy, the vast majority of “state-level poverty-stricken” counties are located in this region as well.^[31]^ Western China should be the key area of the research on village doctors.

In conducting this study, we 1st aimed to update the current status and issues faced by village doctors, particularly in rural western China, and the contributing factors to their job satisfaction. Second, we attempted to fill some gaps in the research field and possibly provide newer evidence for recommendations on village doctor's function, income, and retention for future studies on village doctors in the rural healthcare system, with the ultimate goal of providing references for rural health development and policy formulation. To achieve these 2 objectives, the present study used many subjective measures on the village doctors’ income, social security, work time, and job satisfaction and used also associated factors for job satisfaction based on survey data from village doctors in 30 townships of 2 provinces of western China.

## 2 Methods

### 2.1 Area and sample selection

We employed the multistage stratified sampling method to select the respondents in this study. At the 1st stage, we purposefully selected Gansu Province and Sichuan Province from the country's 12 western provinces. These 2 provinces have similar situations in health services, as profiling the 12 provinces of the western region as a whole. In both provinces, village clinics have been set up for each village (100%); and the number of service providers at the village clinic per thousand rural population in Sichuan and Gansu was the closest to the western average (Sichuan 1.44, Gansu 1.52, and western average 1.45); the total per capita health costs of the 2 provinces was the most alike (1740.81 RMB for Sichuan and 1725.36 RMB for Gansu); and the proportion of the person / number of visits to primary health services to the total number of health institutes was also the closest to the western average (Sichuan 0.34, Gansu 0.38, and western average 0.37). All data were retrieved from China Health and Family Planning Yearbook, 2014.^[32]^

A total of 30 townships from 6 counties in 2 provinces were included in this study. Three counties were chosen from each province by using purposive sampling, based on the geographic distribution of each county and socioeconomic condition at 3 levels from good to poor: Huili, Fushun, and Jiange from Sichuan; and Linze, Yuzhong, and Huining from Gansu (Table 1). Five townships were selected randomly in each county considering different locations, economic conditions, and medical service levels at the same time.

**Table 1:**
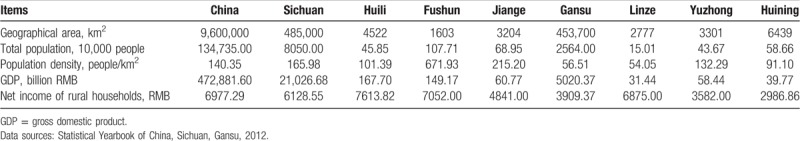
Basic information about 6 counties.

All village doctors in these 30 townships were asked to meet at the township health center and to complete the questionnaire independently. The questionnaire consists of 2 parts, one for village clinics and the other for village doctor. Altogether, 370 village doctors completed the questionnaire survey.

### 2.2 Data collection

Sociology and public health experts and researchers jointly decided the survey plans and tools (e.g., questionnaire). Research assistants and quality controllers were trained for 3 days. Data were collected between July, 2012 and March, 2013. Village doctors were asked to complete the self-administered questionnaire on site. With well-managed on-site coordination, quality control such as on-site inspection, checking, correction, and revisit was conducted by quality controllers.

The questionnaire covered the following aspects of village doctors: demographic characteristics (gender and age), health, education level, major, annual income, and social security, (qualification, working experience, job duties, working hours, and work place, etc). The health measure was defined as absence of any disease by self-reporting. The annual income was made up of 6 parts: service pay from basic medical treatment, essential drug subsidy, subsidy of basic public health services, government subsidy (mainly for some special projects), agricultural products, and other income (e.g., business). Data on the first 4 parts of income came from central and local government, and those on the remaining 2 parts came from respondent's household income. The calculation of income is presented in Section 3.

As a dependent variable, job satisfaction of the village doctors was measured on seven dimensions regarding work condition, service content, technologic level, training content, training times, income, and insurance, which were similar to measures used in other studies.^[33]^ Responses to the questions ranged from “1 = very dissatisfied” to “5 = very satisfied.” The scores of job satisfaction were the total of these items’ scores, thus ranging from 7 to 35. The internal consistency of the job satisfaction items in our study was 0.80 by Kronbach coefficient.

Independent variables included village clinic fund (self-raised, government-funded), equipment (self-purchased, government-supported), gender (male, female), age (years) (≤35, 36–45, 46–55, >55), education level (≤ junior high school, secondary school, >secondary school), health (well, poor), average monthly income (CNY) (≤1000, 1001–1500, 1501–2000, >2000), insurance (yes or no), work years (≤8, 9–10, 11–12, >12), and training times in 2 years (≤5, 6–15, >15).

### 2.3 Data analysis

Data entry was accomplished using Double Entry and Validation through Epidata3.1. To examine associations between covariates and job satisfaction, we 1st performed the univariate analysis to test the differences in job satisfaction scores between different levels within each covariate. Because job satisfaction score was almost normally distributed, statistical significance in the univariate analysis was tested using either *t*-test for covariates with 2 levels or *F*-test for covariates with more than 2 levels. Before fitting multivariate linear regression model to estimate independent effects of covariates on job satisfaction, a variance component model was fitted to the data to test possible cluster effects in the outcome in 30 sampled township health centers. This analysis returned an intra-class correlation coefficient (ICC) of 0.133 or a non-significant proportion of 13.3% variance in the outcome as a result of clustering effects (*P* = .078). Therefore, we used classical multivariate linear regression model to estimate fixed effects of covariates on job satisfaction. A result was considered statistically significant when *P* < .05. Data analyses were performed using SAS 9.4.

### 2.4 Ethics approval

The study design and protocol was approved by the Ethics Committee of West China School of Public Health, Sichuan University.

## 3 Results

### 3.1 General information of village doctors

Village doctors undertook the most primary health work: basic public health services, primary treatment, and rehabilitation of common and frequent diseases.

Among 370 village doctors, the number of men 4 timed that of women. Almost half of the village doctors were between 36 and 45 years old. The majority of them (328/370; 88.6%) had a secondary technical school diploma or under. Only 3 of the 370 village doctors (0.8%) have obtained a college degree. As a result, only 27.5% (102/370) of them had a professional qualification. Many of them (154/370; 41.6%) practiced western medicine, only 61 (16.5%) practiced traditional Chinese medicine (TCM), and 70 (18.9%) practiced the combination of western medicine and TCM (Table 2).

**Table 2:**
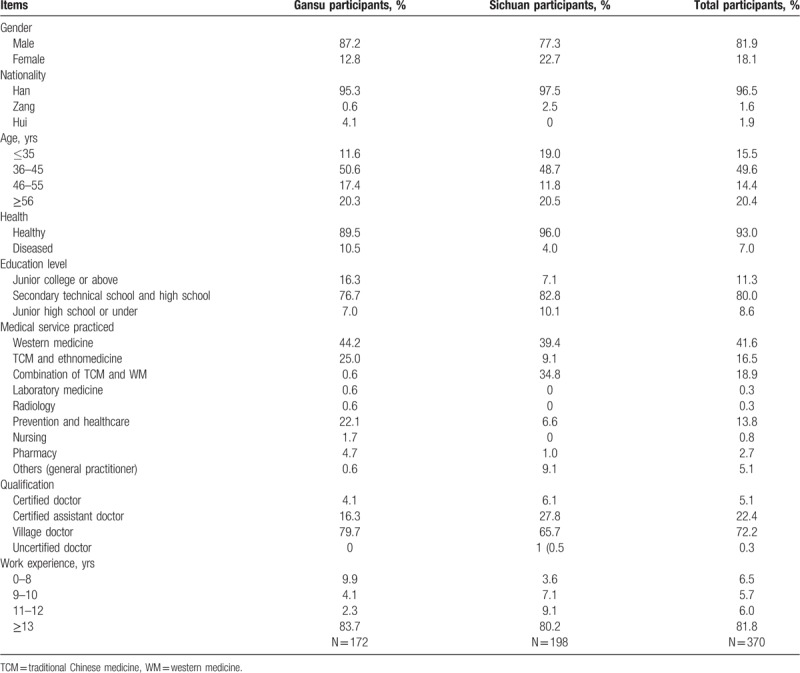
Percentage of general information of village doctors investigated.

### 3.2 Income of village doctors

Village doctors’ income ranged from 1440 RMB to 60,000 RMB as indicated in the questionnaire. The median income of village doctors was 10,000 RMB (interquartile range, 12,000 RMB) in Gansu and 18,000 RMB (interquartile range, 12,000 RMB) in Sichuan.

Based on the policy on subsidies for village doctors from both central and local governments in 2013 and village doctors’ self-reported workload in Village Doctor Questionnaire, we calculated the village doctors’ average annual income by the following equations in an attempt to understand the authenticity of village doctors’ income:

1. Medical treatment subsidy = patient visits/month × 4.5 RMB/person × 12 months + drug subsidy. The average of patient visits is reported by village doctors; and the fee for each medical treatment in village clinics is 5 RMB, with 4.5 RMB reimbursed by new collaborative medical insurance scheme and 0.5 RMB paid by patients.
2. Essential drug subsidy is self-reported.
3. Subsidy on basic public health services = the number of residents in the area where the village doctor is responsible for × 30 RMB/person × 40%/number of clinics in that village. The number of residents covered by service is reported by village doctors; the 30 RMB/person is funded by government for basic public health services per year; and the 40% of basic public health service subsidy goes to village doctors as required by government.
4. Government subsidy (mainly for some special projects) is self-reported.
5. Agricultural income is self-reported.
6. Other sources of income are self-reported.

The sum of the 6-part income makes up a village doctor's annual income.

Table 3 shows the calculated annual income of village doctors.

**Table 3:**
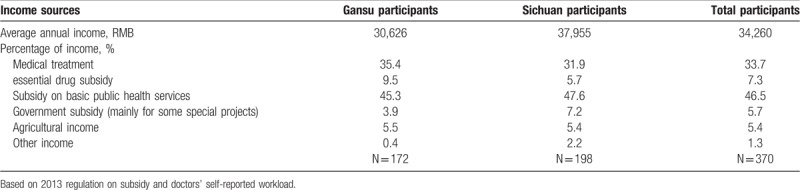
The calculated annual income of village doctors.

### 3.3 Social security of village doctors

Of 370 investigated village doctors, 268 (72.4%) were covered by health insurance. There were 187 (69.8%) village doctors participating in new rural cooperative health insurance, 64 (23.9%) in new rural social old-age insurance, and 7 (2.6%) in commercial insurance.

### 3.4 Working hours of village doctors

The average daily working hours reported by the 370 village doctors were approximately 11.0 hours (interquartile range, 2.0 hours). Up to 56.1% (6.2 hours) of the total working hours were devoted to outpatient services, 26.9% (3.0 hours) to basic public health services, and 17% (1.9 hours) to agriculture, business, and other tasks (Table 4). Village doctors claimed that they provided services to about 200 patients every month on average (250 working days a year) in the clinic, which was equal to seeing 9.6 patients on each working day with each case taking about 20 minutes. They also provided home visits 15 times every month with each case taking 60 minutes. According to our field work observation, the average time spent on each outpatient service was <20 minutes.

**Table 4:**
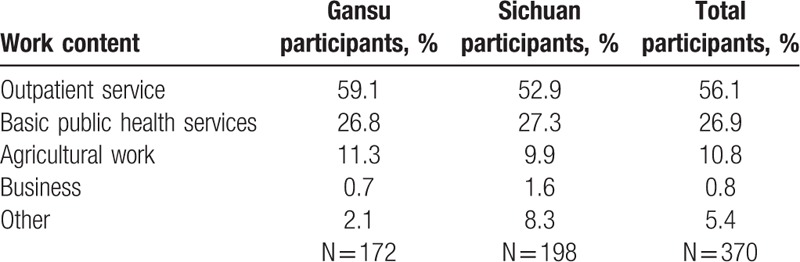
Percentage of working hours distribution reported by village doctors.

### 3.5 Village doctors’ job satisfaction

The overall job satisfaction level of 370 participants was that about 55.4% reported either satisfied or very satisfied with their job. Differences in individual-level characteristics of village doctors’ job satisfaction are summarized in Table 4. Male and older village doctors, as well as those with more training, equipped with government-funded village clinics and township-supported facilities, felt more satisfied with their job (*P* < .05). Education level, health condition with village doctors, as well as monthly income, enrollment in insurance, and work years did not make any significant difference in their job satisfaction.

Multivariate logistic regression analysis was also made to test the determination factors of the village doctors’ job satisfaction. Those who worked with government-funded village clinics (odds ratio [OR] = 3.223, 95% confidence interval [CI]: 1.322–7.857) or government supported facilities (OR = 2.836, 95% CI: 1.174–6.850) were more likely to feel satisfied. Those having poor health (OR = 0.145, 95% CI: 0.029–0.737) and working more than 12 years (OR = 0.219, 95% CI: 0.049–0.980) were more likely to feel less satisfied. On the contrary, the varied gender, age, education level, income, insurance, and training of village doctors did not increase the probability of high job satisfaction (Table 5).

**Table 5:**
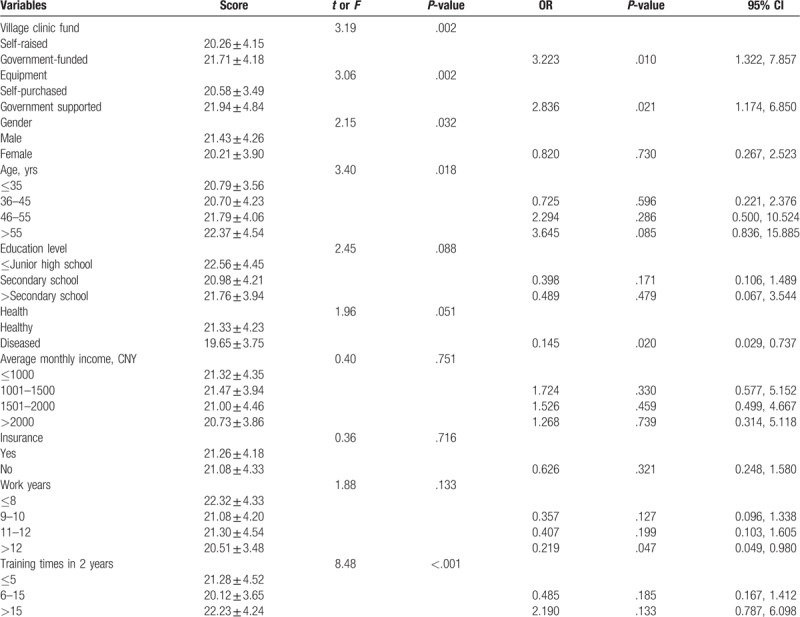
Differences in individual-level characteristics and multivariate logistic regression estimates and variance components of village doctors’ job satisfaction (N = 370).

## 4 Discussion

In this cross-sectional study, income, workload, age, social security, and job satisfaction of 370 village doctors in 2 provinces of Western China were examined. Our findings indicate that government policies in recent years targeting village doctors may have functioned positively on the whole.

### 4.1 Village doctors’ income

Our study has shown that the average annual income of village doctors calculated was at least 30,000 RMB, which would be higher than their self-reported figure. The real income was estimated to be approximately 4 times that of common rural residents (rural per capita net income 8896 RMB in 2013).^[34]^ At the same time, most village doctors were reluctant to provide true information on their agricultural and other income. Therefore, the actual income of village doctors should be more objective if it is considered together with workloads and subsidies condition rather than self-reported only.

Furthermore, to stimulate village doctors to provide primary care health services, the central government pledged per-person subsidies of basic public health service project of 15 RMB in 2009,^[35]^ 20 RMB in 2013, and 55 RMB in 2018,^[36]^ which was increasing year by year. Income of village doctors is also closely related to the support of local governments. Some provinces in China have set up local subsidies for village doctors. For instance, Sichuan Province has implemented the subsidy for essential drugs: 4500 RMB from provincial funding and 2500 RMB from county (city/district) funding for each village clinic per year.^[37]^ In addition, another 1901 RMB was granted for specific purposes per village clinic from the central government.^[38]^ These 3 subsidies totaled approximately 9000 RMB as an extra annual income for village doctors. Central and local governments applied various mechanisms to compensate village doctors, ranging from charge for medical services and basic public health services to the Essential Drugs List allowance and government subsidies. Further investigations and studies on the implementation of these policies at the local level are needed in the future.

While our findings indicated that the local village doctors’ income was improved over what was reported in previous studies, the situation was likely to be different in other areas in China. As a matter of fact, the income gap between economically developed and underdeveloped areas may be immense.

### 4.2 Workload calculation with service items

Different from one previous study,^[20]^ our study found the workload of village doctors not very high, based on their self-reported number of patients and duration of seeing each case, the average daily work time spent on outpatient service was only about 4 hours (9.6 patients × 20 minutes/case + 15 times × 60 minutes/21 days/case = about 235 minutes = about 4 hours), which was just about a 3rd of what they claimed (about 11 hours/day and about half of the statutory 8 hours of work a day).^[39]^ It was actually difficult to distinguish the time spent by village doctors in clinic from that at home, because many of them lived close to the clinic, which enabled them to manage housework and alike during the work time. In addition, we also observed that at least some village doctors’ working time was less than what they reported in the questionnaire. To further consolidate and develop grass-root health services, the government should strengthen investment and define working time for village doctors by the same standard as for other health care and government workers.

### 4.3 Aging of village doctors

The present study showed that aging of village doctors might not be a looming threat to the rural medical service team in China. Among the village doctors in our sample, 65.1% were younger than 45 years old, which was consistent with the finding that 69.4% village doctors were under 46 years of age in Guizhou during the same period.^[22]^ Although these proportions were still lower than the national figure for health workers^[40]^ in 2011, the implementation of the Health System Reform Plan since 2009 has driven the public health cause to develop rapidly. Studies have shown that the age of village doctors gradually presented a younger trend from 2008 to 2012. For instance, village doctors in Chongqing^[41]^ were younger than the national average, and 75.3% village doctors were under 40 years in Puer of Yunnan Province in 2010.^[42]^ Even so, some researchers suggested that aging of village doctors was a serious problem and recommended that efforts be made to improve those situations to stabilize the team.^[43]^ Meanwhile, to retain young village doctors and recruit qualified medical graduates into rural health service work, the central government launched free training of medical students for rural medical and health institutions in 2010, and issued more effective economic and noneconomic policies to encourage young doctors and graduates to engage in rural health.^[44]^ Those policies may have led to an increase in the number of younger village doctors afterwards.

### 4.4 Social security of village doctors

Results of our study showed that village doctors were covered by better social security than other rural residents. Village doctors are typically covered by the same security system as other rural residents, namely the New Rural Cooperative Medical Treatment and the New Rural Social Old-age Insurance, which covered about 90% of village doctors in our investigation. Many local governments have provided village doctors with higher levels of social security such as the Urban and Rural Resident Endowment Insurance. In addition, the central government issued policies to support and guide qualified village doctors to be included in the Employee's Basic Old-age Insurance, indicating that they could enjoy basic life safeguard.^[45]^

### 4.5 Job satisfaction and associated factors

This study found that more than half (55.4%) of village doctors were satisfied with their job, a little higher than the overall job satisfaction level (about 45.4%) reported in China Primary Care Workforce Survey of 2011^[20]^ and notably higher than the result (12.72%) of another survey on village doctors in Jiangxi Province during the new healthcare reforms in 2009.^[21]^ The present study did not find any significant association between job satisfaction and gender, age, education level, average monthly income, insurance, or training times of village doctors, indicating that relevant policy did not work as effectively as intended, partly confirming the research of Tongtong Li.^[46]^ Therefore, approaching the related factors from both the subjective and the objective perspectives of job satisfaction will facilitate development of the rural health service work, by means of policy adjustment. Research results showed that village doctors’ job satisfaction is closely related to the external objective factors, such as government input in funding and facilities and to the length of working time.

### 4.6 Village doctors’ qualification

As an indispensible component of the health service provider and “safeguard” of rural residents’ health, village doctors are obliged to provide the rural population with public health services, basic medical services, etc, on the one hand, and are required to have obtained a diploma from a secondary technical school or above on the other hand.^[45]^ To our delight, results from the present study showed that the majority of village doctors we investigated obtained the village doctor certificate (72.2%) or acquired a diploma from a secondary technical school or above (87.8%).

Nevertheless, village doctors were equipped only with echoscope, sphygmomanometer, thermometer, bio microscope, height and weight scales, mouth gag, tongue depressor, simple respirator, sputum aspirator, debridement package, tourniquet, oxygen bag, and injectors.^[47]^ Without necessary bio/biochemical detectors or iconographic instruments (excluding TCM and ethnomedicine), village doctors are usually challenged with possible misjudgment on acute epidemic diseases, emergent cases, and chronic noninfectious diseases. Research has found that the correct diagnosis rate of village clinics was only 26%.^[48]^

On the contrary, it has been the goal of the Chinese government to implement universal coverage of public health services, improve service quality, and ultimately equalize access to basic public health services for both urban and rural residents.^[49]^ This, together with the change in rural public health service demands, has further defined the importance and challenges of grass-root public health services and made us reflect on the qualifications of and requirements for village doctors in providing higher quality basic public health services.

To summarize, results of the present study suggest that the government human resource policies may have played a positive role in rural China. At the national level, the number of village doctors in China totaled about 1 million and has remained stable in recent years.^[50]^ At the provincial level, the number of village doctors in Sichuan Province grew by 6% in 3 consecutive years after the New Medical Reform was launched.^[51]^ Findings of this study are consistent with some international studies focusing on the rural health system. For example, Saini et al reported that shortage of infrastructure and salary were perceived as potential barriers to a career of health professionals in India's rural health, and concluded that both problems need specific interventions from the government.^[52]^ Shankar found that government investment in improving working conditions in rural Nepal would assist rural communities to attract and retain doctors.^[53]^ Additionally, the Global Conference on Primary Health Care in 2018, co-sponsored by WHO, UNICEF, and the Government of Kazakhstan, adopted the Declaration of Astana, which reiterates the responsibility of government in primary health care and in the support for human resources for health with decent work, appropriate compensation, training, and retention.^[54]^ Evidences in China, such as those reported in our study, may shed light on the development of rural health human resources as a global issue, and particularly in developing countries.

### 4.7 Limitations

Despite the findings, our study has some limitations. First, although the internal consistency of measurement tools is good, some report bias might have been presented with the self-reported data of job satisfaction. Second, although the provinces selected in the study basically represent the typical economic and health conditions in western China, the selection of a wider geographical area would give the conclusions of the study more robustness and external validity. Third, the cross-sectional nature of this study indicates only correlation among the variables. It would be more useful to investigate the causality of the variables in future longitudinal studies, such as how healthcare policies play a role in the village doctors’ job satisfaction. For a more comprehensive understanding of rural health services, studies on township- and county-level medical health services should be included for comparison.

## 5 Conclusion

Our study highlighted findings different from other studies on situations of village doctors in western China. It revealed that, at least in the investigated areas, the team of village doctors was generally stable, and no extreme staff turnover or aging problem was observed. Village doctors enjoyed much higher average annual income and better social security than other rural residents. The working time of most village doctors was within the national legal working hour standards. Job satisfaction of village doctors had improved with the development of reform policies since 2009. Further analysis showed that village doctor's job satisfaction is closely related to health, work years, and government input in funding and facilities. It is noteworthy that the situation reflected in the survey is directly related to relevant policies. Government policies and financial support are the key to the sustainable and stable development of rural healthcare as evidenced by, village doctors’ satisfaction in particular.

Our study findings suggest that the government should take into full consideration the imbalanced socioeconomic development and differentiated demands of public health services across the nation, and formulate more specific measures targeting local conditions. For this reason, further strengthening government policies and financial support, including installing and upgrading rural health facilities and improve village doctors’ income and social security policies, would not only accelerate the improvement of rural health service quality, but also help maintain village doctors’ satisfaction and stimulate their working enthusiasm.

The qualification of village doctors should be shifted from medical treatment to public health service, and the regional health planning should act in compliance with market demands and regional disparity. Moreover, these findings about the current status of village doctors within the Chinese health care reform experience could be beneficial to other developing countries in developing community and rural health services.

## Acknowledgment

The authors thank all respondents and the support from Sichuan and Gansu Provincial Centers for Disease Prevention and Control, and the health officials in these 2 provinces. The authors also thank Prof Dongtao Lin of Sichuan University for copyediting this manuscript.

## Author contributions

**Conceptualization:** Qi Zhang, Xiao Ma.

**Data curation:** Qi Zhang, Jiayan Chen, Tao Mao, Cong Zhang, Xiao Ma.

**Formal analysis:** Jiayan Chen, Min Yang, Jay Pan, Lin Yue, Tao Mao, Cong Zhang.

**Funding acquisition:** Qi Zhang, Xiao Ma.

**Investigation:** Qi Zhang, Jiayan Chen, Tao Mao.

**Project administration:** Qi Zhang, Xiao Ma.

**Supervision:** Xiao Ma.

**Writing – original draft:** Qi Zhang.

**Writing – review & editing:** Qi Zhang, Min Yang, Jay Pan, Xiaoping Li, Lin Yue, Yuan Huang.

Xiao Ma orcid: 0000-0001-8987-6639.
